# Vibration-Induced Errors in MEMS Tuning Fork Gyroscopes with Imbalance

**DOI:** 10.3390/s18061755

**Published:** 2018-05-29

**Authors:** Xiang Fang, Linxi Dong, Wen-Sheng Zhao, Haixia Yan, Kwok Siong Teh, Gaofeng Wang

**Affiliations:** 1 The Key Laboratory of RF Circuits and System of Ministry of Education, College of Electronic and Information, Hangzhou Dianzi University, Hangzhou 310018, China; fangxiang@hdu.edu.cn (X.F.); wsh.zhao@gmail.com (W.-S.Z.); gaofeng@hdu.edu.cn (G.W.); 2 State Key Laboratory of Functional Materials for Informatics, Chinese Academy of Sciences, Shanghai 200050, China; 3 Department of Mechanics, School of Information Engineering, Hangzhou Dianzi University, Hangzhou 310018, China; 4 State Key Laboratory for Manufacturing Systems Engineering, Xi’an Jiaotong University, Xi’an 710049, China; 5 Department of Mechanics, School of Engineering, San Francisco State University, San Francisco, CA 94132, USA; kwok.siong@gmail.com

**Keywords:** vibration, TFG, simulation, imbalance, error output, coupling

## Abstract

This paper discusses the vibration-induced error in non-ideal MEMS tuning fork gyroscopes (TFGs). Ideal TFGs which are thought to be immune to vibrations do not exist, and imbalance between two gyros of TFGs is an inevitable phenomenon. Three types of fabrication imperfections (i.e., stiffness imbalance, mass imbalance, and damping imbalance) are studied, considering different imbalance radios. We focus on the coupling types of two gyros of TFGs in both drive and sense directions, and the vibration sensitivities of four TFG designs with imbalance are simulated and compared. It is found that non-ideal TFGs with two gyros coupled both in drive and sense directions (type CC TFGs) are the most insensitive to vibrations with frequencies close to the TFG operating frequencies. However, sense-axis vibrations with in-phase resonant frequencies of a coupled gyros system result in severe error outputs to TFGs with two gyros coupled in the sense direction, which is mainly attributed to the sense capacitance nonlinearity. With increasing stiffness coupled ratio of the coupled gyros system, the sensitivity to vibrations with operating frequencies is cut down, yet sensitivity to vibrations with in-phase frequencies is amplified.

## 1. Introduction

With the advancement of microelectromechanical system (MEMS) technology [1], gyroscopes are progressing towards low cost, compact, low power, light weight, and high-performance. The performance of MEMS vibratory gyroscopes (MVGs) has achieved great improvement in resolution, sensitivity, and bandwidth [2,3,4,5,6]. Therefore, applications of MVGs are growing rapidly in the fields of consumer, automobile, industry, navigation, and military devices. MVGs operate based on the energy transfer between two gyro vibration modes caused by the Coriolis effect [7] and usually have a high quality factor (Q-factor) ranging from 45 in air [8] to tens of thousands in vacuum [9]. Higher Q factor improves the resolution and sensitivity of MVGs. Meanwhile, it also amplifies the amplitudes of displacements or forces caused by external vibration at certain frequencies, and results in increased output errors [10]. These output errors, known as “vibration sensitivity” are unpredictable, and difficult to minimize with better electronics circuits design.

To avoid vibration-induced output errors, tuning fork gyroscopes (TFGs) are designed to eliminate linear vibrations output as a common mode signal by differential sensing. TFGs have two identical masses that vibrate out of phase [11], which cancel common-mode noises and double the amplitude of the output signal compared to conventional MVGs. In general, TFGs are considered to be insensitive to vibrations [12,13]. However, previous works found that linear vibrations along the sense direction of TFGs still induce error outputs [14,15]. Yoon et al. [16] reported three causes of vibration sensitivity in ideal TFGs: (1) the asymmetric electrostatic force along the sense direction; (2) the asymmetric electrostatic force along the drive direction; and (3) nonlinearity in the capacitance of parallel plate sense electrodes. These three causes, which may appear in special situations and some TFG designs, were analyzed and compared in three TFG designs [10,17,18] by Matlab and Simulink. However, the conclusion was based on ideal TFGs, which do not exist because of technological limitations. Earlier studies found that the sense direction vibration sensitivity of TFGs with stiffness imbalance can be reduced by increasing the decoupling ratio and coupled stiffness ratio [19,20]. However, only vibration with operating frequency and sense stiffness imbalance were studied. Moreover, the coupling between two TFG gyros was not considered in previous works.

In this paper, we conducted a detailed analysis of TFG vibration sensitivity, considering three types of imbalance caused by fabrication defects. A two degrees-of-freedom (DOFs) coupled gyros model and a two DOFs uncoupled gyros model are established, and their theoretical dynamics are studied when both anti-phase forces and in-phase vibration accelerations are applied to two of their gyros. Sense capacitive nonlinearity leading to small output errors induced by vibrations with non-operating frequencies is included in this work. And the nomenclature of variables used in the work is given in Table 1. The vibration sensitivities of four TFG designs with different coupling types between two gyros are compared though simulation. To compare fairly, all designs considered in this paper were assumed to be operating ideally (except for the previously-discussed imbalance), and other effects caused by quadrature errors, mismatch during fabrication, or asymmetric forces are excluded in this work.

## 2. Theoretical Study on the Vibration Response of the Ideal TFG

**Figure 1:**
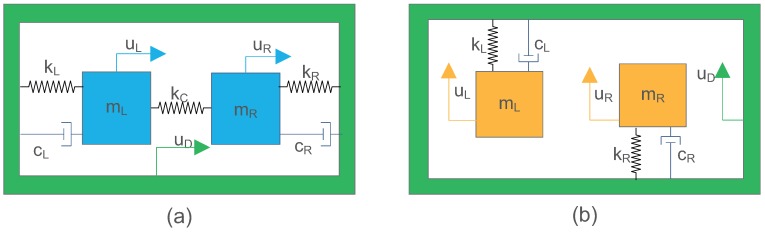
Two degrees-of-freedom (DOFs) model of a tuning fork gyroscope (TFG) experiencing external vibration. (**a**) TFG model with two gyros coupled on the drive or sense axis. (**b**) TFG model with two gyros uncoupled on the drive or sense axis.

The structure of a TFG can be simplified into two DOFs models as shown in Figure 1. In general, TFGs consists of two gyros with vertical vibrating modes: drive mode and sense mode. The two gyros can be coupled together in each mode direction to separate the operating frequency from other model frequencies (see Figure 1a), or uncoupled for simple structure and high sensitivity (see Figure 1b).

When the TFG is not in operation, the response dynamics of the coupled left and right gyros suffering external vibration are governed by:

for the left gyro:
  (1) $$m_{L}{\ddot{u}}_{L}+c_{L}\left({\dot{u}}_{L}-{\dot{u}}_{D}\right)+k_{L}\left(u_{L}-u_{D}\right)-k_{C}\left(u_{R}-u_{L}\right)=0,$$
for the right gyro:
  (2) $$m_{R}{\ddot{u}}_{R}+c_{R}\left({\dot{u}}_{R}-{\dot{u}}_{D}\right)+k_{R}\left(u_{R}-u_{D}\right)+k_{C}\left(u_{R}-u_{L}\right)=0.$$

With consideration of internal force $F_{L},F_{R}$, the displacement dynamics of the coupled left and right gyros can be expressed in matrix form as:

(3) $$\left[\begin{array}{cc} m_{L} & 0\\ 0 & m_{R} \end{array}\right]\ddot{v}+\left[\begin{array}{cc} c_{L} & 0\\ 0 & c_{R} \end{array}\right]\dot{v}+\left[\begin{array}{cc} k_{L}+k_{C} & -k_{C}\\ -k_{C} & k_{R}+k_{C} \end{array}\right]=\left[\begin{array}{c} -m_{L}a_{v}+F_{L}\\ -m_{R}a_{v}+F_{R} \end{array}\right],$$

where $v=\left[\begin{array}{c} v_{L}\\ v_{R} \end{array}\right]$, and $v_{L}=u_{L}-u_{D}$ and $v_{R}=u_{R}-u_{D}$ are the displacements of two gyros relative to the device.

For an ideal TFG, take $F_{L}=-F_{R}=F_{0}\sin\left(\omega_{0}t\right)$ and $a_{v}=a_{0}\sin\left(\omega_{v}t+\phi \right)$ into Equation (3). Through the mode superposition method, the steady responses of two gyros can be given by:

(4) $$v=\left[\begin{array}{c} \frac{-a_{0}\beta_{1}}{\omega_{1}^{2}}\sin\left(\omega_{v}t+\phi -\phi_{1}\right)+\frac{F_{0}\beta_{2}}{m\omega_{2}^{2}}\sin\left(\omega_{0}-\phi_{2}\right)\\ \frac{-a_{0}\beta_{1}}{\omega_{1}^{2}}\sin\left(\omega_{v}t+\phi -\phi_{1}\right)-\frac{F_{0}\beta_{2}}{m\omega_{2}^{2}}\sin\left(\omega_{0}-\phi_{2}\right) \end{array}\right]=\left[\begin{array}{c} v_{a}+v_{F}\\ v_{a}-v_{F} \end{array}\right],$$

where $\omega_{1}=\sqrt{\frac{k}{m}}$ and $\omega_{2}=\sqrt{\frac{k+2k_{C}}{m}}$ denote the first-order (in-phase) and second-order (anti-phase) resonant angular frequencies, $\beta_{i}=\frac{1}{\sqrt{{\left(1-\lambda_{i}^{2}\right)}^{2}+{\left(2\xi_{1}\lambda_{i}\right)}^{2}}}$ are the magnification factors of amplitude, $\lambda_{1}=\frac{\omega_{v}}{\omega_{1}}$ and $\lambda_{2}=\frac{\omega_{0}}{\omega_{2}}$ are the frequency ratios, the phase angles $\phi_{i}=\arctan\frac{2\xi_{1}\lambda_{i}}{1-\lambda_{i}^{2}}$, and $\xi_{i}=\frac{c}{2m\omega_{i}}$ are the damping ratios, i=1,2.

For the uncoupled gyros model, the response dynamics to external vibration of the uncoupled left and right gyros when the TFG is not in operation are governed by:

for the left gyro:
  (5) $$m_{L}{\ddot{u}}_{L}+c_{L}\left({\dot{u}}_{L}-{\dot{u}}_{D}\right)+k_{L}\left(u_{L}-u_{D}\right)=0,$$
for the right gyro:
  (6) $$m_{R}{\ddot{u}}_{R}+c_{R}\left({\dot{u}}_{R}-{\dot{u}}_{D}\right)+k_{R}\left(u_{R}-u_{D}\right)=0.$$

Steady responses of the two gyros of an ideal TFG in operation can be calculated, with $F_{L}=-F_{R}=F_{0}\sin\left(\omega_{0}t\right)$ and $a_{v}=a_{0}\sin\left(\omega_{v}t+\phi \right)$:

(7) $$v=\left[\begin{array}{c} \frac{-a_{0}\beta_{1}}{\omega^{2}}\sin\left(\omega_{v}t+\phi -\phi_{1}\right)+\frac{F_{0}\beta_{2}}{m\omega^{2}}\sin\left(\omega_{0}t-\phi_{2}\right)\\ \frac{-a_{0}\beta_{1}}{\omega^{2}}\sin\left(\omega_{v}t+\phi -\phi_{1}\right)-\frac{F_{0}\beta_{2}}{m\omega^{2}}\sin\left(\omega_{0}t-\phi_{2}\right) \end{array}\right]=\left[\begin{array}{c} v_{a}+v_{F}\\ v_{a}-v_{F} \end{array}\right],$$

where $v=\left[\begin{array}{c} v_{L}\\ v_{R} \end{array}\right]$, and $v_{L}=u_{L}-u_{D}$ and $v_{R}=u_{R}-u_{D}$ are the displacements of two gyros relative to the device. $\omega =\sqrt{\frac{k}{m}}$ is the angular resonant frequency, $\beta_{i}=\frac{1}{\sqrt{{\left(1-\lambda_{i}^{2}\right)}^{2}+{\left(2\xi_{1}\lambda_{i}\right)}^{2}}}$ denote the magnification factors of amplitude, the frequency ratios are $\lambda_{1}=\frac{\omega_{v}}{\omega }$ and $\lambda_{2}=\frac{\omega_{0}}{\omega }$, the phase angles are $\phi_{i}=\arctan\frac{2\xi_{1}\lambda_{i}}{1-\lambda_{i}^{2}}$, and $\xi_{i}=\frac{c}{2m\omega_{i}}$ are the damping ratios, i=1,2.

In summary, it is clear that the displacement differences on both sense and drive axes will not change while suffering external vibrations for an ideal MEMS TFG. This is the advantage of TFGs compared to single mass MEMS gyroscopes.

## 3. Sense Capacitance Nonlinearity

Capacitive detection is known to offer several benefits compared to other sensing techniques, especially its ease of implementation. Capacitive detection does not require the integration of a special material, which makes them compatible with state-of-the-art fabrication processes. They also provide good DC response and noise immunity, high sensitivity, low drift, and low temperature sensitivity. It is advantageous for TFGs’ double masses and anti operating model structure to apply differential capacitive detection.

**Figure 2:**
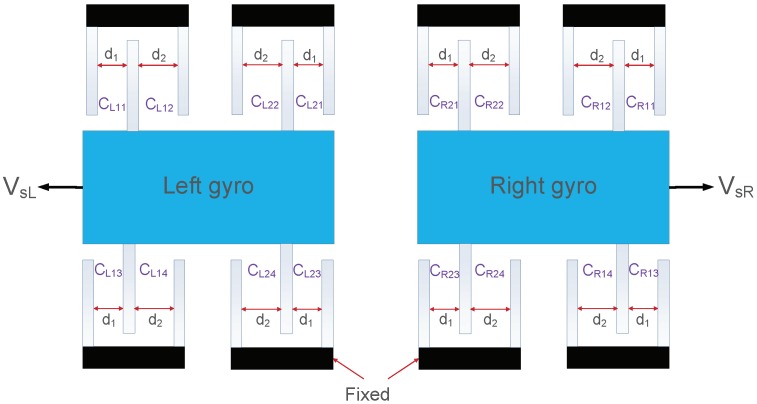
Differential configuration of variable-gap capacitors on the sense axis of a TFG with initial unequal sense capacitance gaps $d_{1}$ and $d_{2}$. The capacitance value of each capacitor is given as $C_{L1i}/C_{L2i}/C_{R1i}/C_{R2i}$, i=1,2,3,4.

However, the parallel-plate sensing mechanism displays a nonlinear behavior between sense capacitance and the sense-axis displacement. A simplified model of the differential capacitive sensing of a TFG is shown in Figure 2. Several groups of capacitive electrodes are symmetrically placed on two opposing sides of two sensing masses. When the TFG is in operation, the displacements of two gyro masses will be transferred to the capacitance change. If the TFG is not in operation, the initial capacitance of two gyro masses can be expressed as:

(8) $$C_{0}=C_{L}=C_{R}=C_{L1}+C_{L2}=C_{R1}+C_{R2}=4\varepsilon A_{s}\left(\frac{1}{d_{1}}+\frac{1}{d_{2}}\right).$$

It is supposed that no external vibration exists, or that the amplitude of vibration displacement is much smaller than the sense capacitance gaps ($d_{1}$, $d_{2}$). The final output capacitance of a TFG derived in Appendix A can be similarly expressed as:

(9) $$C_{o}≅4\varepsilon A_{s}\left(\frac{1}{d_{1}}+\frac{1}{d_{2}}\right)\left(v_{sL}-v_{sL}\right)\left(\frac{1}{d_{1}}-\frac{1}{d_{2}}\right),$$

where $v_{sL}$ and $v_{sR}$ denote the displacements of two gyros along the sense direction.

The demodulation system recovers only the output signals whose frequencies are at or near the gyro’s operating frequency. With the approximate calculation lists in Appendix A, the error capacitance that cannot be filtered by demodulating and rotation-related capacitance with considering $v_{sL}=V_{0}\sin\left(\omega_{0}t\right)+V\sin\left(\omega_{v}t+\varphi \right)$ and $v_{sR}=-V_{0}\sin\left(\omega_{0}t\right)+V\sin\left(\omega_{v}t+\varphi \right)$ are given by:

(10) $$C_{v}≅3C_{0}\left(\frac{1}{d_{1}}-\frac{1}{d_{2}}\right)V^{2}V_{0}\sin\left(\omega_{0}t\right)\left(\frac{1}{d_{1}^{2}}-\frac{1}{d_{2}^{2}}\right),$$

(11) $$C_{r}≅2C_{0}\left(\frac{1}{d_{1}}-\frac{1}{d_{2}}\right)V_{0}\sin\left(\omega_{0}t\right).$$

Vibration performance is determined by the ratio η of the gyro’s vibration sensitivity over the gyro’s rotation sensitivity:

(12) $$\eta =\frac{C_{v}}{C_{r}}=\frac{3}{2}V^{2}\left(\frac{1}{d_{1}^{2}}-\frac{1}{d_{2}^{2}}\right).$$

From Equation (12), it can be found that designers should decrease the Q value of the TFG or decrease the sense capacitances’ gap difference and increase gap distances to reduce the vibration error outputs caused by sense capacitance nonlinearity. Either method has to lower the TFG’s rotation sensitivity.

## 4. Theoretical Study of the Vibration Response of a Non-Ideal TFG

Due to the limitations of current fabrication technology, the mass, stiffness, and damping of left and right gyros are not perfectly identical. Three types of fabrication imperfections are discussed in the following subsections. First, the two resonant angular frequencies of the two DOFs systems shown in Figure 1 are given by:

coupled gyros:
  (13) $$\omega_{1,2}^{2}=\frac{1}{2}\left(\frac{k_{L}+k_{C}}{m_{1}}+\frac{k_{R}+k_{C}}{m_{2}}\right)\pm \frac{1}{2}\sqrt{{\left(\frac{k_{L}+k_{C}}{m_{1}}-\frac{k_{R}+k_{C}}{m_{R}}\right)}^{2}+\frac{4k_{C}^{2}}{m_{L}m_{R}}},$$
uncoupled gyros:
  (14) $$\begin{array}{c} \omega_{1}=\sqrt{\frac{k_{L}}{m_{L}}},\omega_{2}=\sqrt{\frac{k_{R}}{m_{R}}}. \end{array}$$

### 4.1. Stiffness Imbalance

With stiffness imbalance ratio (SIR) $a_{k}$ in the calculation, the displacement dynamics of the two coupled gyros shown in Figure 1a are given in Appendix B and resonant angular frequencies of the system are calculated from:

(15) $$\omega_{1,2}^{2}=\frac{\left(1+a_{k}+2b\right)\pm \sqrt{{\left(1-a_{k}\right)}^{2}+4b^{2}}}{2m}k,\left(\omega_{1}<\omega_{2}\right).$$

For most TFG designs, the operating frequency is set to be at or near the anti-phase resonant frequency (i.e., $\omega_{0}=\omega_{2}$) of the system. If no vibration exists, the error ratio defined by the ratio of error displacements difference and ideal displacements difference is given by:

(16) $$\rho_{k}≅-\frac{{\left(I_{k2}+1\right)}^{2}}{2+2I_{k2}^{2}}.$$

Since only vibrations whose angular frequencies are near $\omega_{1,2}$ lead to large displacements and error signals at $\omega_{1}$ will be filtered by the system. Besides, the influence of vibrations is much larger than the internal forces (detailed in Appendix B). Hence, with only the vibrations at $\omega_{2}$ considered, the error ratio defined as the ratio of error displacements difference and ideal error displacements difference is given as:

(17) $$\rho_{k}≅\left(\frac{1}{2}+\frac{1}{2I_{k2}}\right)\frac{ma_{0}}{F_{0}}\frac{\cos(\omega_{2}t+\phi )}{\cos(\omega_{2}t)},$$

where $I_{k2}=\frac{1-a_{k}-\sqrt{{\left(1-a_{k}\right)}^{2}+4b^{2}}}{2b}$ is the anti-phase mode factor (close to −1 when negligible imbalance exists) of the coupled gyros system, and ϕ is the phase difference between vibration acceleration and operating forces.

The displacement dynamics of the uncoupled gyros system shown in Figure 1b with (SIR) $a_{k}$ are derived in Appendix C. The error displacements difference ratio when vibrations with ideal resonant frequency ($\frac{\omega_{1}}{2\pi }$) exist is given by:

(18) $$\rho_{k}≅\left(\frac{1}{J_{k}}\sin\phi_{k}-\frac{1}{2}\right)\frac{ma_{0}}{F_{0}}\frac{\cos(\omega_{1}t+\phi )}{\cos(\omega_{1}t)}+\left(\frac{1}{J_{k}}\sin\phi_{k}-\frac{1}{2}\right),$$

where $\omega_{1}=\sqrt{\frac{k}{m}}$ and $\omega_{2}=\sqrt{\frac{a_{k}k}{m}}$ are the resonant angular frequencies of left and right gyros of uncoupled gyros system, coefficient $J_{k}=\sqrt{{\left(a_{k}-1\right)}^{2}/\xi_{1}^{2}+4}$, the phase angle $\phi_{k}=\arctan\frac{2\xi_{1}\sqrt{a_{k}}}{a_{k}-1}$, the damping ratio $\xi_{1}=\frac{c}{2m\omega_{1}}$, and ϕ is the phase difference between vibration acceleration and operating forces.

### 4.2. Mass Imbalance

With the mass imbalance ratio (MIR) $a_{m}=\frac{1}{a_{m}^{\prime }}$ in the calculation, the displacement dynamics of the two coupled gyros shown in Figure 1a are given in Appendix D, and the resonant angular frequencies of the system can be obtained from:

(19) $$\omega_{1,2}^{2}=\frac{\left(1+a_{m}^{\prime }\right)\left(1+b\right)\pm \sqrt{{\left(1-a_{m}^{\prime }\right)}^{2}{\left(1+b\right)}^{2}+4a_{m}^{\prime }b^{2}}}{2m}k,\left(\omega_{1}<\omega_{2}\right).$$

The error displacements difference ratio is given by (calculated in Appendix D):

When no vibration exists and operating forces are driving forces, that is, along the drive direction ($F_{L}=F_{d}\sin\left(\omega_{2}t\right)$, $F_{R}=-F_{d}\sin\left(\omega_{2}t\right)$):

(20) $$\rho_{m}≅\frac{{\left(I_{m2}-1\right)}^{2}}{2+2a_{m}I_{m2}^{2}}-1;$$

when no vibration exists and operating forces are Coriolis forces, that is, along the sense direction ($F_{L}=ma_{co}\sin\left(\omega_{2}t\right)$, $F_{R}=-a_{m}ma_{co}\sin\left(\omega_{2}t\right)$):

(21) $$\rho_{m}≅\left(-\frac{1}{2}-\frac{\left(1+a_{m}\right)I_{m2}}{2+2a_{m}I_{m2}^{2}}\right);$$

when vibration with $\omega_{2}$ exists and the effect of operating forces are neglected:

(22) $$\rho_{m}≅\left(1-\frac{1-a_{m}}{2\sqrt{a_{m}}}+\frac{1}{\sqrt{a_{m}}I_{m2}}\right)\frac{\alpha }{2}\frac{\cos(\omega_{2}t+\phi )}{\cos(\omega_{2}t)},$$

where $I_{m2}=\frac{\left(1-1/a_{m}\right)\left(1+b\right)-\sqrt{{\left(1-1/a_{m}\right)}^{2}{\left(1+b\right)}^{2}+4b^{2}/a_{m}}}{2b}$ is the anti-phase mode factor (close to −1 when little imbalance exists) of the system, α is the force ratio given by $ma_{0}/F_{0}$, and ϕ is the phase difference between vibration acceleration and operating forces.

The displacement dynamics of the two uncoupled gyros shown in Figure 1b with (MIR) $a_{m}$ are derived in Appendix E. The error displacements difference ratio for vibrations with anti-phase resonant frequencies is given by:

When operating forces are driving forces, that is, along the drive direction ($F_{L}=F_{d}\sin\left(\omega_{2}t\right)$, $F_{R}=-F_{d}\sin\left(\omega_{2}t\right)$):

(23) $$\rho_{m}≅\left(\frac{a_{m}}{J_{m}}\sin\phi_{m}-\frac{1}{2}\right)\frac{ma_{0}}{F_{d}}\frac{\cos(\omega_{1}t+\phi )}{\cos(\omega_{1}t)}+\left(\frac{1}{J_{m}}\sin\phi_{m}-\frac{1}{2}\right);$$

when operating forces are Coriolis forces, that is, along the sense direction ($F_{L}=ma_{co}\sin\left(\omega_{2}t\right)$, $F_{R}=-a_{m}ma_{co}\sin\left(\omega_{2}t\right)$):

(24) $$\rho_{m}≅\left(\frac{a_{m}}{J_{m}}\sin\phi_{m}-\frac{1}{2}\right)\frac{a_{0}}{a_{co}}\frac{\cos(\omega_{1}t+\phi )}{\cos(\omega_{1}t)}+\left(\frac{a_{m}}{J_{m}}\sin\phi_{m}-\frac{1}{2}\right),$$

where $\omega_{1}=\sqrt{\frac{k}{m}}$ and $\omega_{2}=\sqrt{\frac{k}{a_{m}m}}$ are the resonant angular frequencies of the left and right gyros of the uncoupled gyro system, coefficient $J_{m}=\sqrt{{\left(a_{m}-1\right)}^{2}/\xi_{1}^{2}+4}$, the phase angle $\phi_{m}=\arctan\frac{2\xi_{1}\sqrt{a_{m}}}{1-a_{m}}$, the damping ratio $\xi_{1}=\frac{c}{2m\omega_{1}}$, and ϕ is the phase difference between vibration acceleration and operating force.

### 4.3. Damping Imbalance

Damping imbalance only influences the amplitudes and phases angle of gyro displacements. The resonant frequencies of two gyros system are constant regardless of damping imbalance. The displacement dynamics of two coupled gyros with damping imbalance ratio (DIR) $a_{c}$ are derived in Appendix F in Laplace form. The error seems to be very small compared with other imbalances, and the error displacements difference ratio for the two uncoupled gyros system considering $a_{c}$ can be expressed by (Appendix G):

(25) $$\rho_{c}=\left(\frac{1}{2a_{c}}-\frac{1}{2}\right)\frac{a_{0}}{F_{0}}\frac{\cos(\omega t+\phi )}{\cos(\omega t)}+\left(\frac{1}{2a_{c}}-\frac{1}{2}\right),$$

where $\omega =\sqrt{\frac{k}{m}}$ is the resonant angular frequency of the left and right gyros of the uncoupled gyros system, and ϕ is the phase difference between vibration acceleration and operating force.

### 4.4. Summary

As discussed previously, the dynamics and error displacements difference ratios of two gyros systems are similar for stiffness and mass imbalance. It can be found that the anti-phase mode factors of coupled gyros systems with stiffness or mass imbalance are influenced by the imbalance ratio and the coupled stiffness ratio. With stiffness or mass IRs far away from 1, anti-phase mode factors become far away from 1 and error displacements difference ratios increase. By increasing the coupled stiffness ratio, error displacements difference ratios are reduced for the same stiffness or mass imbalance ratio. For uncoupled gyros systems with stiffness and mass imbalance, the error displacements difference ratios are directly related to imbalance ratios and the Q factor ($Q≅\frac{1}{2\xi }$). For a TFG with Q = 50, the error displacements difference ratios of uncoupled gyros systems are much larger than those of coupled gyros systems with the same stiffness and mass imbalance regardless of vibrations. Moreover, it is more severe for TFGs with uncoupled gyros as Q factors become higher. Damping imbalance also induces error outputs to TFGs, and it seems to be much less influential than stiffness and mass imbalance.

## 5. Models and Parameters

A TFG consists of two gyros which are designed to vibrate out of phase. Each gyro contains two modes vertical to each other (i.e., drive mode and sense mode). TFGs are divided into four groups by coupling each gyro’s two mode masses; that is, (1) CP type—TFGs that have coupled sense and drive masses on each gyro [17], (2) DS type—TFGs that have decoupled sense and drive masses with an anchored sense mass on each gyro [10], (3) DD type—TFGs that have decoupled sense and drive masses with an anchored drive mass on each gyro [18,21], (4) fully decoupled type—TFGs that have fully decoupled sense and drive masses with drive and sense masses anchored on each gyro [22,23]. They respond differently to external vibration for several reasons, as discussed in a previous study [19]. Only imbalance and sense capacitance nonlinearity which exist in each TFG are considered herein. Drive capacitance, quadrature error, and other reasons are neglected.

**Figure 3:**
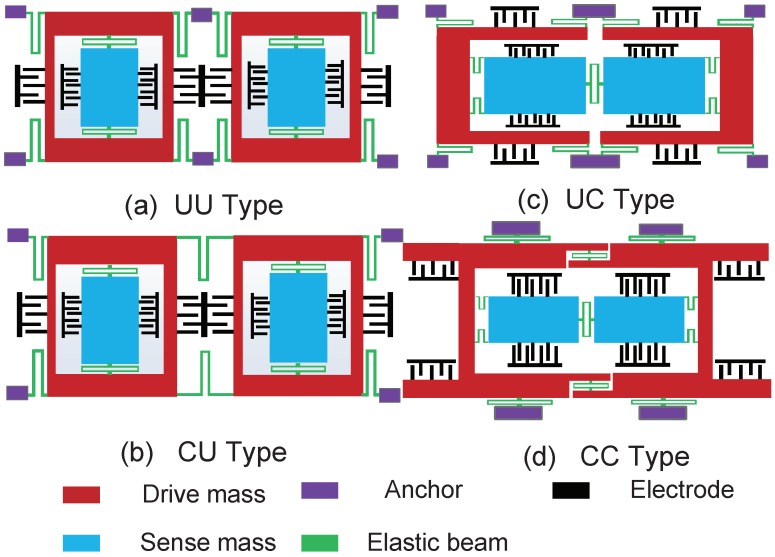
Four different designs of DD-type TFGs. (**a**) UU-type TFG: design that has two gyros uncoupled in both drive and sense directions, (**b**) CU-type TFG: design that has two gyros coupled in the drive direction and uncoupled in the sense direction, (**c**) UC-type TFG: design that has two gyros uncoupled in the drive direction and coupled in the sense direction, (**d**) CC-type TFG: design that has two gyros coupled in both drive and sense directions.

From the theoretical study above, it is known that coupling types between two gyros of TFGs influence the error displacements ratio differently. For fair comparison, four designs of DD-type TFGs are modeled as CP shown in Figure 3. They are named as UU type—a design that has two gyros uncoupled in both drive and sense directions; CU type—a design that has two gyros coupled in the drive direction and uncoupled in the sense direction; UC type—a design that has two gyros uncoupled in the drive direction and coupled in the sense direction; and CC type—a design that has two gyros coupled in both drive and sense directions.

**Figure 4:**
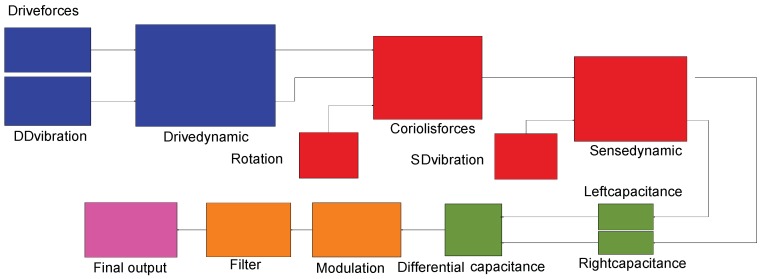
Simulink block model of a TFG in operation suffering external vibrations, details not shown were created by the subsystem.

The model parameters are listed in Table 2. To achieve a fair comparison, all types of TFGs are assumed to have the same parameters. The Simulink model shown in Figure 4 was designed to operate as real a TFG and the details were created by the subsystem, which is not shown in the figure. The model consists of (1) the driving force, (2) the dynamic of the sense and drive masses, (3) the vibration sources and vibration-induced forces, (4) the differential capacitive output, and (5) the demodulation system. The demodulation system recovers only signals whose frequencies are at/near the gyro’s resonant frequency, as in the case of real matched-mode gyroscopes. All the simulated results were obtained with the same demodulation, and other adjustment methods were not considered.

## 6. Simulation and Discussion

In this section, the vibration sensitivity of TFGs is exhibited by the simulated responses of their models to a constant normal rotation signal (angular velocity: $100^{\circ }/s$) and in-plane vibration. The left gyro of each TFG is supposed to be flawless, and the imbalance ratio (IR) is defined by the ratio of the properties of the right gyro to the left gyro. Different imbalance types studied with parametric IR include stiffness, mass, or damping imbalance (SIM, MIM, or DIM, respectively). The vibration sensitivity of four TFG designs and TFGs with different coupled stiffness ratios (CRs) were compared to find methods of reducing vibration sensitivity. The operation frequencies of all TFG systems remained at 10 kHz. Vibrations with resonant frequencies were the main focus, since vibrations with other frequencies induce much smaller displacements.

**Figure 5:**
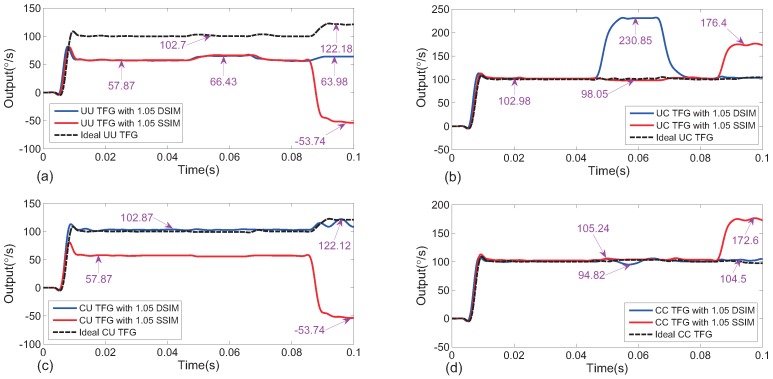
Time-varying outputs of four TFG types—(**a**) UU-type, (**b**) UC-type, (**c**) CU-type, (**d**) CC-type—with or without stiffness imbalance (IR = 1.05) in one direction when no vibration exists, when only the drive axis vibration works (0.04–0.06 s), and when only sense axis vibration works (0.08–0.10 s). All frequencies and phases were assumed to be 10 kHz and 0, respectively. The vibration amplitudes were set to be 20g, but for CU-type and UU-type, they were set to 2g for the normal operation of TFGs. DSIM: drive stiffness imbalance; SSIM: sense stiffness imbalance.

Figure 5 demonstrates responses from four ideal TFG designs with the same stiffness imbalance and in drive or sense direction with or without vibration. Consistent with the study in the previous section, error outputs were time-related, and thus outputs which were the farthest away from an ideal output are marked approximately. Through comparison of the responses of four ideal TFGs, it is seen that TFGs with gyros uncoupled on the sense axis were more sensitive to vibration along the sense direction and about 22% error output corresponding to 2*g*, amplitudes existed because of sense capacitance nonlinearity. As for error outputs induced only by imbalance, the UU-type TFG with drive or sense stiffness imbalance and the CU-type TFG with sense stiffness imbalance produced error outputs with about $-42^{\circ }$/s. However, for the UC TFG, the error was much smaller. Error amplitude differences of drive-axis displacements led to not exactly antisymmetric Coriolis acceleration, and resulted in amplitude differences of sense-axis displacements and final error outputs . As discussed previously, the error displacements difference ratios for uncoupled gyros induced only by stiffness imbalance were much larger than that for coupled gyros. Considering vibrations, it could be found that outputs with vibrations and stiffness imbalance in the sense direction were almost not influenced by coupling types in the drive direction. In contrast, the values of error outputs with vibrations and stiffness imbalance in the drive direction were are enlarged by an uncoupled gyros structure on the sense axis compared to a coupled gyros structure. The simulation results also revealed that TFGs with two gyros uncoupled in one direction showed larger same-axis vibration and stiffness imbalance sensitivity. As shown in Figure 5a,d, error outputs with vibrations and stiffness on the sense axis were larger than that on the drive axis with the same coupling structure. According to the contrast of UU-type TFG with UC-type TFG and CU-type TFG, it is clear that the uncoupled gyros structures are more sensitive to vibrations with operating frequencies compared with coupled gyros structures on the same axis.

Simulated outputs of different TFGs with different types of imbalance on the drive or sense axis before and during vibration consistent with the direction of imbalance are given in Table 3 and Table 4. The influence of mass imbalance was similar to that of stiffness imbalance, and damping imbalance was less influential than both of them on vibration sensitivity. As with the conclusion we make for Figure 5, UU-type TFGs were the most sensitive to vibrations with operating frequencies and imbalance and CC-type TFGs were the most insensitive.

**Figure 6:**
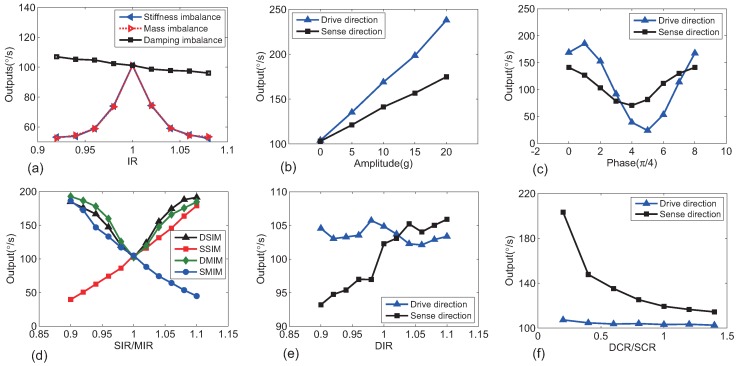
(**a**) Outputs of UU-type TFGs with different imbalance and IR when no vibration existed. (**b**–**e**) Outputs of UC-type TFGs with different imbalance type and ratio and different vibrations in the same direction: (**b**) Variable amplitudes ($a_{0}$), (**c**) Variable vibration acceleration phases (ϕ), (**d**) Different SIR or MIR ($a_{k},a_{m}$), (**e**) Different DIR ($a_{c}$). (**f**) Outputs of CC-type TFGs with different CR in the case that SIM and vibration is in the same direction. Default parameters were supposed as: $a_{0}=10$ g, ϕ=0, $a_{k}=1.05$, $a_{m}=1$, $a_{c}=1$, b=0.5, and f=10 kHz.

The outputs of UU-type TFGs with different imbalance and IRs in the sense direction are shown in Figure 6a. In keeping with the previous theoretical study, the error ratios without vibration for uncoupled gyros system were approximatively inversely proportional to IR for small damping imbalance and $\sqrt{{\left(IR-1\right)}^{2}}$ for small stiffness and mass imbalance. Relationships between amplitudes or phases of vibrations with operating frequencies and UC-type TFG outputs are depicted in Figure 6b,c, respectively, and stiffness imbalance IR = 1.05 in the same direction with vibration was considered. Amplitudes were found to be linearly related to the outputs. The variable phases changed both absolute values and the directions of changing trends of error outputs and the relation curves were approximately trigonometric. Influences of different IRs were simulated and are shown in Figure 6d,e. It could be found that absolute values of error outputs increased as IRs moved far away from 1 (ideal TFGs) for stiffness and mass imbalance. UC-type TFGs consist of two gyros uncoupled in the drive direction and coupled in the sense direction. Since uncoupled gyros systems respond to vibration and imbalance differently than coupled gyros systems, relationships between IRs and vibration error outputs in the drive direction or the sense direction are different. On the drive axis of UC-type TFGs, vibration error outputs were inversely proportional to $\sqrt{{\left(IR-1\right)}^{2}}$ for small stiffness and mass imbalance. However, error outputs were similarly linearly related for IRs of slight stiffness and mass imbalance with vibration error outputs of UC-type TFGs on the sense axis. The influence of damping imbalance was much less than that of mass and stiffness imbalance. Therefore, error outputs caused by imbalanced operating displacements and sense capacitance nonlinearity cannot be ignored compared with vibration error outputs. They were all influenced by the damping imbalance ratio and the outputs changed with DIR irregularly, as shown in Figure 6e. For coupled gyro systems, coupled stiffness ratios (CRs) were also related to the error outputs. Keeping the operating frequency and ideal anti-phase resonant frequency at 10 kHz, outputs of CC-type TFGs with different CRs in two directions are given in Figure 6f. Stiffness imbalance with IR =1.05 and vibration are supposed to be in the same direction. It is seen that as CR increased, the error outputs were reduced, and the effect of increasing CRs became insignificant when CRs became larger.

**Figure 7:**
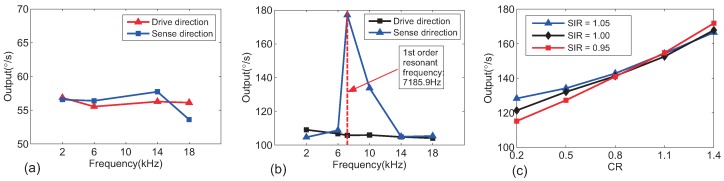
Simulated outputs of (**a**) UU-type TFGs and (**b**) CC-type TFGs with different frequency vibrations. Stiffness imbalance (IR = 1.05) is supposed to be in the same direction as vibration and phases and amplitudes were set to 0 and 10*g* except for 3*g* for the sense-axis first-order resonant frequency in (b) for the TFGs’ normal operation. (**c**) Vibration outputs of CC-type TFGs with different CR suffering vibration with in-phase resonant frequency in the sense direction. Amplitudes, phase, and frequency were set to *g*, 0, and at the in-phase resonant frequencies (listed in Table 5). Situations with or without sense stiffness imbalance were all considered.

Figure 7a shows simulated outputs of a UU-type TFG with different-frequencies vibrations and stiffness imbalance (IR = 1.05). Vibration outputs in the same direction with vibration whose frequency is at operating frequency (10 kHz) are not shown because they are too large. For a CC type TFG in the same situation, the results are given in Figure 7b. It was found that vibrations with non-operating and non-resonant frequencies induced smaller error outputs than vibration with operating frequencies, but error outputs existed because of imbalance and sense capacitance nonlinearity. However, for CC-type TFGs, vibrations in the sense direction with in-phase resonant frequencies induced very large error outputs because large error in-phase resonant displacements were produced, and it was similar to 10 kHz vibrations in the uncoupled gyros systems. As the amplitudes of resonant displacements were inversely proportional to the square of the resonant frequencies, the error outputs were inversely proportional to CR to maintain anti-phase resonant frequencies, as shown in Figure 7c.

## 7. Conclusions

The vibration sensitivity of different TFG designs was studied theoretically and with simulation considering sense capacitance nonlinearity and three types of imbalance induced by fabrication defects. It is commonly thought that TFGs are vibration-insensitive because they are designed with two identical gyros that vibrate out-of-phase to cancel common-mode noises. However, ideal TFGs do no exist due to technological limitations, and imbalance cannot be avoided. A two DOFs coupled gyros model and a two DOFs uncoupled gyros model are established corresponding to different TFG designs. Approximate calculations and dynamic simulations were used to obtain their output dynamics with imbalance when both anti-phase forces and in-phase accelerations are applied to their two masses. It was found that damping imbalance was less influential on vibration sensitivity compared with stiffness and mass imbalances. TFGs with two gyros coupled in both drive and sense directions (CC-type TFGs) were more insensitive to vibrations with frequencies near the TFG operating frequency due to the smallest error displacements. The operating frequency vibration insensitivity could be enhanced by increasing the stiffness coupled ratio (CR). However, vibrations with frequencies close to the in-phase resonant frequencies of two coupled gyros along the sense direction resulted in devastating error outputs for CC-type TFGs, mainly because of sense capacitance nonlinearity, and the error outputs were approximately proportional to the square of CRs. Therefore, avoiding resonant frequencies of TFG systems and vibration isolation are two effective external methods to reduce TFGs’ vibration sensitivity. A CC-type TFG with low quality factor, proper sense coupled stiffness ratio, and large variable-gap capacitors gaps or applying sensing variable-area capacitors may be a better choice for vibration environments.

## Figures and Tables

**Table 1:** Nomenclature of variables in this work.

**Force-related**	
$F_{L}/F_{R}$	Operating force (driving or Coriolis force) of the left/right gyro of a TFG
$a_{v}$	External vibration acceleration
$a_{co}$	Coriolis acceleration amplitude: response of TFG to rotation in normal direction
$F_{d}$	Driving force amplitude
$F_{0}/a_{0}$	Amplitude of $F_{L}$ and $F_{R}$ in ideal TFG/Amplitude of $a_{v}$
$\omega_{0}$	Operating angular frequency
$\omega_{v}$	Vibration angular frequency
$\omega_{1}/\omega_{2}$	First/second resonant angular frequency of 2-DOFs model
ϕ	Phase of the external vibration acceleration
**Displacement-related**	
$u_{L}/u_{R}$	Total absolute displacement of the left/right gyro of a TFG
$v_{L}/v_{R}$	Total displacement of the left/right gyro of a TFG relative to the device
$u_{D}$	Total absolute displacement of the device
$v_{a}/v_{F}$	Displacement caused by operating force / external vibration
*v*	$\left[\begin{array}{c} v_{L}\\ v_{R} \end{array}\right]$
$v_{e}/v_{i}$	Error/ideal displacements difference
**TFG structure-related**	
$m_{L}/m_{R}/m$	Mass of left gyro/right gyro/left or right gyro of ideal TFG
$k_{L}/k_{R}/k$	Stiffness of left gyro mass/right gyro mass/left or right gyro mass of ideal TFG
$k_{c}$	Coupling stiffness between left and right gyro mass
$c_{L}/c_{R}/c$	Damping of left gyro mass/right gyro mass/left or right gyro mass of ideal TFG
$a_{k}/a_{m}/a_{c}/a_{m}^{\prime }$	Stiffness/mass/damping imbalance ratio (IR)/$\frac{1}{a_{m}}$
*b*	Coupling stiffness ratio (CR)
**Capacitance-related**	
$C_{L}/C_{R}$	Total capacitance value of the left/right gyro
$C_{0}$	Value of $C_{L}$ and $C_{R}$ of ideal TFG not in operation
$A_{s}$	Sense capacitance overlapping area
$d_{1},d_{2}$	Initial sense capacitance gaps
$C_{L1i}/C_{L2i}$, i=1,2,3,4	Capacitance value of each capacitor of left gyro shown in Figure 2
$C_{R1i}/C_{R2i}$, i=1,2,3,4	Capacitance value of each capacitor of right gyro shown in Figure 2
$C_{L1}/C_{L2}$	Given by $\left(C_{L11}+C_{L12}+C_{L13}+C_{L14}\right)$/$\left(C_{L21}+C_{L22}+C_{L23}+C_{L24}\right)$
$C_{R1}/C_{R2}$	Given by $\left(C_{R11}+C_{R12}+C_{R13}+C_{R14}\right)$/$\left(C_{R21}+C_{R22}+C_{R23}+C_{R24}\right)$
$\Delta C_{L}/\Delta C_{R}$	Differential capacitive readouts of the left/right gyro, given by $C_{L1}-C_{L2}$/$C_{R1}-C_{R2}$
$C_{o}$	Final capacitance output of a TFG, given by $\Delta C_{L}-\Delta C_{R}$
$C_{v}/C_{r}$	Final capacitance output caused by external vibration/rotation
ε	Permittivity
**Other subscripts**	
d/s	Subscripts indicating drive/sense mode or direction
k/m/c	Subscripts indicating stiffness/mass/damping-related
1/2	First/second resonant frequency-related displacement or coefficient
**Calculation-related**	
$I_{k1}/I_{k2}$	In-phase/anti-phase modal factor of coupled gyros system with stiffness imbalance
$I_{m1}/I_{m2}$	In-phase/anti-phase modal factor of coupled gyros system with mass imbalance
α	Force ratio given by $ma_{0}/F_{0}$ or $a_{0}/a_{co}$
$\rho_{k}/\rho_{m}/\rho_{c}$	Error displacements difference ratio, given by $\frac{v_{e}}{v_{i}}$, with stiffness/mass/damping imbalance

**Table 2:** Model parameters in the simulation.

Parameters	Value	Parameters	Value
Resonant frequency	10 kHz	Q-factor	50
Drive mass	2 μg	Sense mass	0.8 μg
Driving force amplitude	0.2 μN	Drive damping coefficient	$2.5\times 10^{-6}$ Ns/m
Sense damping coefficient	$1.0\times 10^{-6}$ Ns/m	Drive-mode siffness	3.95 N/m
Sense-mode stiffness	2.26 N/m	Sense capacitance overlapping area	$1\times 10^{-9}$ m${}^{2}$
Initial total sense capacitance	$5.31\times 10^{-13}$ F	Sense comb number	80
Sense capacitance gaps	1, 2 μm	Low-pass filter cut-off frequency	100 Hz

**Table 3:** Outputs of different TFGs with drive stiffness/mass/damping imbalance (DSIM/DMIM/DDIM) and sense stiffness/mass/damping imbalance (SSIM/SMIM/SDIM). The IRs were all set to 1.05 and no vibration existed.

TFG Type	Simulated Output of TFGs (∘/s)					
	DSIM	DMIM	DDIM	SSIM	SMIM	SDIM
**UU-Type**	57.13	56.55	96.28	56.70	57.55	95.68
**UC-Type**	103.95	98.73	102.27	102.98	95.99	101.48
**CU-Type**	105.06	93.36	102.44	56.70	57.48	95.69
**CC-Type**	101.02	103.01	102.42	102.98	95.98	101.82

**Table 4:** Outputs of different TFGs experiencing vibration with DSIM/DMIM/DDIM and SSIM/SMIM/SDIM. The IRs were all set to 1.05 and vibration directions were supposed to be the same with imbalance. Vibration acceleration frequencies and phases were 10 kHz and 0, respectively. The amplitudes were set at 10g, but for UU-type and CU-type with SSIM, SMIM, and SDIM, they were set to 2g for TFGs’ proper operation.

TFG Type	Simulated Output of TFGs (∘/s)					
	DSIM	DMIM	DDIM	SSIM	SMIM	SDIM
**UU-Type**	106.01	104.09	104.02	−54.56	−50.44	112.04
**UC-Type**	168.82	156.94	107.07	141.12	68.29	102.86
**CU-Type**	106.44	92.92	104.49	−54.46	−50.44	112.04
**CC-Type**	102.41	107.24	102.58	141.12	68.29	102.86

**Table 5:** In-phase resonant frequencies of CC-type TFGs in the sense direction with different CRs and SIRs used in Figure 7c.

	CR	In-Phase Sense Axis Resonant Frequency (Hz)				
SIR		0.2	0.5	0.8	1.1	1.4
**1.05**		8556.5	7158.9	6278.8	5659.6	5193.6
**1**		8451.5	7071.1	6201.7	5590.2	5129.9
**0.95**		8345.2	6982.1	6123.7	5519.9	5065.4

## Appendix A

The rotation component and vibration component of output capacitance as the gyros are in motion on the sense axis are derived. When the TFG is in operation and responsive to angular velocity, differential capacitive readouts of the left and right gyros are given by:

(A1) $$\begin{array}{cc} \Delta C_{L} & =C_{L1}-C_{L2}=2\varepsilon A_{s}\left(\frac{1}{d_{1}-v_{sL}}+\frac{1}{d_{2}+v_{sL}}\right)-2\varepsilon A_{s}\left(\frac{1}{d_{1}+v_{sL}}+\frac{1}{d_{2}-v_{sL}}\right)\\ & =4\varepsilon A_{s}v_{sL}\left(\frac{1}{d_{1}^{2}-v_{sL}^{2}}-\frac{1}{d_{2}^{2}-v_{sL}^{2}}\right) \end{array},$$

(A2) $$\begin{array}{cc} \Delta C_{R} & =C_{R1}-C_{R2}=2\varepsilon A_{s}\left(\frac{1}{d_{1}-v_{sR}}+\frac{1}{d_{2}+v_{sR}}\right)-2\varepsilon A_{s}\left(\frac{1}{d_{1}+v_{sR}}+\frac{1}{d_{2}-v_{sR}}\right)\\ & =4\varepsilon A_{s}v_{sR}\left(\frac{1}{d_{1}^{2}-v_{sR}^{2}}-\frac{1}{d_{2}^{2}-v_{sR}^{2}}\right) \end{array}.$$

Therefore, the final differential capacitance output of TFG:

(A3) $$C_{o}=\Delta C_{L}-\Delta C_{R}≅\frac{4\varepsilon A_{s}v_{sL}}{d_{1}^{2}}\left(1+\frac{v_{sL}^{2}}{d_{1}^{2}}\right)+\frac{4\varepsilon A_{s}v_{sR}}{d_{2}^{2}}\left(1+\frac{v_{sR}^{2}}{d_{2}^{2}}\right)-\frac{4\varepsilon A_{s}v_{sR}}{d_{1}^{2}}\left(1+\frac{v_{sR}^{2}}{d_{1}^{2}}\right)-\frac{4\varepsilon A_{s}v_{sL}}{d_{2}^{2}}\left(1+\frac{v_{sL}^{2}}{d_{2}^{2}}\right),$$

where $v_{sL}$ and $v_{sR}$ denote the sense axis displacements of the two gyros. If $v_{sL},v_{sR}\ll d_{1}<d_{2}$, $1+\frac{v_{sL}^{2}}{d_{i}^{2}}≅1$ and $1+\frac{v_{sL}^{2}}{d_{i}^{2}}≅1$. Then:

(A4) $$C_{o}≅4\varepsilon A_{s}\left(\frac{1}{d_{1}}+\frac{1}{d_{2}}\right)\left(v_{sL}-v_{sL}\right)\left(\frac{1}{d_{1}}-\frac{1}{d_{2}}\right)=A\left(v_{sL}-v_{sR}\right).$$

Supposing rotation is constant, let $v_{sL}=V_{0}\sin\left(\omega_{0}t\right)+V\sin\left(\omega_{v}t+\varphi \right)$ and $v_{sR}=-V_{0}\sin\left(\omega_{0}t\right)+V\sin\left(\omega_{v}t+\varphi \right)$, the capacitance output can be transferred to:

(A5) $$C_{o}≅2AV_{0}\sin\left(\omega_{0}t\right)+A\left(B_{1}+B_{2}+B_{3}\right),$$

(A6) $$B_{1}=3V^{2}V_{0}\sin\left(\omega_{0}t\right)\left(\frac{1}{d_{1}^{2}}-\frac{1}{d_{2}^{2}}\right),$$

(A7) $$B_{2}=-\frac{3}{2}V^{2}V_{0}\sin\left(\left(\omega_{0}+2\omega \right)t+2\varphi \right)\left(\frac{1}{d_{1}^{2}}-\frac{1}{d_{2}^{2}}\right),$$

(A8) $$B_{3}=-\frac{3}{2}V^{2}V_{0}\sin\left(\left(\omega_{0}-2\omega \right)t-2\varphi \right)\left(\frac{1}{d_{1}^{2}}-\frac{1}{d_{2}^{2}}\right).$$

In the case of the same TFG, *A* is a constant, and $B_{1},B_{2}$ and $B_{3}$ are the third power term of displacement with different angular frequencies; that is, $\omega_{0}$, $\omega_{0}+2\omega_{v}$, and $\omega_{0}-2\omega_{v}$. Since in most situations, $AB_{2}$ and $AB_{3}$ are filtered by the modulation system of TFG, the output capacitance related to rotation and vibration can be written as: $C_{r}=2AV_{0}\sin\left(\omega_{0}t\right)$ and $C_{v}=AB_{1}$. Hence, the vibration performance ratio is achieved in Equation (12).

## Appendix B

With the parameters SIR $a_{k}$, $F_{L}=-F_{R}=F_{0}\sin\left(\omega_{0}t\right)$, and $a_{v}=a_{0}\sin\left(\omega_{v}t+\phi \right)$ in consideration, displacements dynamics in Equation (3) are derived by modal superposition method. The displacements of two coupled gyros are shown as follows:

(A9) $$\omega_{1,2}^{2}=\frac{\left(1+a_{k}+2b\right)\pm \sqrt{{\left(1-a_{k}\right)}^{2}+4b^{2}}}{2m}k,\left(\omega_{1}<\omega_{2}\right),$$

(A10) $$v=\left[\begin{array}{c} v_{a1}+v_{F1}+v_{a2}+v_{F2}\\ I_{k1}v_{a1}+I_{k1}v_{F1}+I_{k2}v_{a2}+I_{k2}v_{F2} \end{array}\right]=\left[\begin{array}{c} v_{L}\\ v_{R} \end{array}\right],$$

(A11) $$I_{k1}=\frac{1-a_{k}+\sqrt{{\left(1-a_{k}\right)}^{2}+4b^{2}}}{2b},$$

(A12) $$I_{k2}=\frac{1-a_{k}-\sqrt{{\left(1-a_{k}\right)}^{2}+4b^{2}}}{2b},$$

(A13) $$v_{ai}=\frac{-1-I_{ki}}{1+I_{ki}^{2}}\frac{a_{0}}{\omega_{i}^{2}\sqrt{{\left(1-\lambda_{ai}^{2}\right)}^{2}+{\left(2\xi_{i}\lambda_{ai}^{2}\right)}^{2}}}\sin\left(\omega_{v}t+\phi -\phi_{ai}\right)=\frac{-1-I_{ki}}{1+I_{ki}^{2}}v_{a0i},$$

(A14) $$v_{Fi}=\frac{1-I_{ki}}{1+I_{ki}^{2}}\frac{F_{0}}{m\omega_{i}^{2}\sqrt{{\left(1-\lambda_{Fi}^{2}\right)}^{2}+{\left(2\xi_{i}\lambda_{Fi}\right)}^{2}}}\sin\left(\omega_{0}t-\phi_{Fi}\right)=\frac{1-I_{ki}}{1+I_{ki}^{2}}v_{F0i},$$

where $I_{k1}>0$ and $I_{k2}<0$ denote modal shape factors used in the modal superposition method with stiffness imbalance considered whose absolute value are very near 1 and $I_{k1}I_{k2}=-1$, $\omega_{1}$, and $\omega_{2}$ are the resonant angular frequencies of the system, the frequency ratios $\lambda_{ai}=\frac{\omega_{v}}{\omega_{i}}$ and $\lambda_{Fi}=\frac{\omega_{0}}{\omega_{i}}$, $\xi_{i}=\frac{c}{2m\omega_{i}}$ are the damping ratios, the phase angles $\phi_{ai}=\arctan\frac{2\xi_{i}\lambda_{ai}}{1-\lambda_{ai}^{2}}$, $\phi_{Fi}=\arctan\frac{2\xi_{i}\lambda_{Fi}}{1-\lambda_{Fi}^{2}}$, i=1,2. $v_{a0i}$ and $v_{F0i}$ are vibration- and internal force-related displacement terms.

The displacements difference can be calculated through Equations (A9)–(A14):

(A15) $$v_{L}-v_{R}=\frac{I_{k1}^{2}-1}{1+I_{k1}^{2}}v_{a01}+\frac{{\left(I_{k1}-1\right)}^{2}}{1+I_{k1}^{2}}v_{F01}+\frac{I_{k2}^{2}-1}{1+I_{k2}^{2}}v_{a02}-\frac{{\left(I_{k2}+1\right)}^{2}}{1+I_{k2}^{2}}v_{F02}+2v_{F02}=v_{e}+2v_{F02}.$$

For most TFG designs, the operating frequencies are set to be at or near the anti-phase resonant frequencies ($\frac{\omega_{2}}{2\pi }$) of the system. Therefore, the $v_{F01}$ term can be ignored. If no vibration exists, the error displacement difference ratio is given by:

(A16) $$\rho_{k}≅-\frac{{\left(I_{k2}+1\right)}^{2}}{2+2I_{k2}^{2}}.$$

Since $I_{k1}^{2}-1=\left(I_{k1}-1\right)\left(I_{k1}+1\right)\gg {\left(I_{k1}-1\right)}^{2},I_{k2}^{2}-1\gg {\left(I_{k2}+1\right)}^{2}$ and ${\left(1-I_{k1}\right)}^{2}\ll 2I_{k1}$, ${\left(1+I_{k2}\right)}^{2}\ll \left|2I_{k2}\right|$ when external vibration displacements are large enough compared to operating displacements. Error displacements difference $v_{e}$ in Equation (A15) is approximately calculated by:

(A17) $$v_{e}=\left(1-\frac{2}{{\left(1-I_{k1}\right)}^{2}+2I_{k1}}\right)v_{a01}+\left(1-\frac{2}{{\left(1+I_{k2}\right)}^{2}-2I_{k2}}\right)v_{a02}≅\left(1-\frac{1}{I_{k1}}\right)v_{a01}+\left(1+\frac{1}{I_{k2}}\right)v_{a02}.$$

Supposing $\omega_{0}=\omega_{v}=\omega_{2}$:

(A18) $$v_{a01}=\frac{a_{0}}{\omega_{1}^{2}\sqrt{{\left(1-\lambda^{2}\right)}^{2}+{\left(2\xi_{1}\lambda^{2}\right)}^{2}}}\sin\left(\omega_{2}t+\phi -\phi_{1}\right),$$

(A19) $$v_{a02}=\frac{-a_{0}}{\omega_{2}^{2}2\xi_{2}}\cos\left(\omega_{2}t+\phi \right),$$

(A20) $$v_{F02}=\frac{-F_{0}}{2m\omega_{2}^{2}\xi_{2}}\cos\left(\omega_{2}t\right).$$

For a TFG with high Q, the $v_{a01}$ can be neglected compared with $v_{a02}$. Hence,

(A21) $$\begin{array}{c} v_{i}=2v_{F02}=\frac{-F_{0}}{m\omega_{2}^{2}\xi_{2}}\cos\left(\omega_{2}t\right), \end{array}$$

(A22) $$\begin{array}{c} v_{e}≅\left(1+\frac{1}{I_{k2}}\right)\frac{-a_{0}}{\omega_{2}^{2}2\xi_{2}}\cos\left(\omega_{2}t+\phi \right), \end{array}$$

where the frequency ratio $\lambda =\frac{\omega_{2}}{\omega_{1}}$, $\xi_{i}=\frac{c}{2m\omega_{i}}$ are the damping ratios, the phase angle $\phi_{1}=\arctan\frac{2\xi_{1}\lambda }{1-\lambda^{2}}$, i=1,2. Equation (17) is obtained by $v_{e}/v_{i}$.

## Appendix C

The steady responses of two uncoupled gyros are derived with stiffness imbalance. The displacements of two uncoupled gyros with SIR $a_{k}$, $F_{L}=-F_{R}=F_{0}\sin\left(\omega_{0}t\right)$, and $a_{v}=a_{0}\sin\left(\omega_{v}t+\phi \right)$ are given by:

(A23) $$\begin{array}{c} \omega_{1}=\sqrt{\frac{k}{m}},\omega_{2}=\sqrt{\frac{a_{k}k}{m}}, \end{array}$$

(A24) $$\begin{array}{c} v=\left[\begin{array}{c} \frac{-a_{0}\beta_{a1}}{\omega_{1}^{2}}\sin\left(\omega_{v}t-\phi_{a1}\right)+\frac{F_{0}\beta_{F1}}{m\omega_{1}^{2}}\sin\left(\omega_{0}t-\phi_{F1}\right)\\ \frac{-a_{0}\beta_{a2}}{\omega_{2}^{2}}\sin\left(\omega_{v}t-\phi_{a2}\right)-\frac{F_{0}\beta_{F2}}{m\omega_{2}^{2}}\sin\left(\omega_{0}t-\phi_{F2}\right) \end{array}\right]=\left[\begin{array}{c} v_{L}\\ v_{R} \end{array}\right], \end{array}$$

where $\beta_{ai}=\frac{1}{\sqrt{{\left(1-\lambda_{ai}^{2}\right)}^{2}+{\left(2\xi_{i}\lambda_{ai}\right)}^{2}}}$ and $\beta_{Fi}=\frac{1}{\sqrt{{\left(1-\lambda_{Fi}^{2}\right)}^{2}+{\left(2\xi_{i}\lambda_{Fi}\right)}^{2}}}$ are the magnification factors of amplitude, $\omega_{1}$ and $\omega_{2}$ are the resonant angular frequencies of the system, the frequency ratios $\lambda_{ai}=\frac{\omega_{v}}{\omega_{i}}$ and $\lambda_{Fi}=\frac{\omega_{0}}{\omega_{i}}$, $\xi_{i}=\frac{c}{2m\omega_{i}}$ are the damping ratios, the phase angles $\phi_{ai}=\arctan\frac{2\xi_{i}\lambda_{ai}}{1-\lambda_{ai}^{2}}$, $\phi_{Fi}=\arctan\frac{2\xi_{i}\lambda_{Fi}}{1-\lambda_{Fi}^{2}}$, i=1,2.

If $\omega_{v}=\omega_{0}=\omega_{1}=\frac{1}{\sqrt{a_{k}}}\omega_{2}$, the displacements of two gyros can be written as:

(A25) $$v=\left[\begin{array}{c} \frac{a_{0}}{2\xi_{1}\omega_{1}^{2}}\cos\left(\omega_{1}t+\phi \right)-\frac{F_{0}}{2\xi_{1}m\omega_{1}^{2}}\cos\left(\omega_{1}t\right)\\ \frac{-a_{0}}{J_{k}\xi_{1}\omega_{1}^{2}}\sin\left(\omega_{1}t+\phi -\phi_{1}\right)-\frac{F_{0}}{J_{k}\xi_{1}m\omega_{1}^{2}}\sin\left(\omega_{1}t-\phi_{1}\right) \end{array}\right]=\left[\begin{array}{c} v_{L}\\ v_{R} \end{array}\right],$$

(A26) $$J_{k}=\frac{a_{k}}{\xi_{1}}\sqrt{{\left(1-\frac{\omega_{1}^{2}}{\omega_{2}^{2}}\right)}^{2}+{\left(2\xi_{2}\frac{\omega_{1}}{\omega_{2}}\right)}^{2}}=\sqrt{{\left(a_{k}-1\right)}^{2}/\xi_{1}^{2}+4},$$

where the phase angle $\phi_{1}=\arctan\frac{2\xi_{1}\sqrt{a_{k}}}{a_{k}-1}$, which is close to 90 degrees, and the damping ratio $\xi_{1}=\frac{c}{2m\omega_{1}}$. Therefore, substitute $\sin\left(\omega_{1}t+\phi -\phi_{1}\right)≅-\cos\left(\omega_{1}t\right)\sin\phi$ to Equation (A25) and the displacements difference is given as:

(A27) $$v_{L}-v_{R}≅\left(\frac{1}{2\xi_{1}}-\frac{1}{J_{k}\xi_{1}}\sin\phi_{k}\right)\frac{a_{0}}{\omega_{1}^{2}}\cos\left(\omega_{1}t+\phi \right)-\left(\frac{1}{2\xi_{1}}+\frac{1}{J_{k}\xi_{1}}\sin\phi_{k}\right)\frac{F_{0}}{m\omega_{1}^{2}}\cos\left(\omega_{1}t\right).$$

So, the error displacements difference and ideal displacements difference can be calculated by:

(A28) $$v_{e}≅\left(\frac{1}{2\xi_{1}}-\frac{1}{J_{k}\xi_{1}}\sin\phi_{k}\right)\frac{a_{0}}{\omega_{1}^{2}}\cos\left(\omega_{1}t+\phi \right)+(\frac{1}{2\xi_{1}}-\frac{1}{J_{k}\xi_{1}}\sin\phi_{k}\frac{1}{2\xi_{1}})\frac{F_{0}}{m\omega_{1}^{2}}\cos\left(\omega_{1}t\right),$$

(A29) $$v_{i}=\frac{-F_{0}}{\xi_{1}m\omega_{1}^{2}}\cos\left(\omega_{1}t\right),$$

and Equation (18) is obtained by Equation (A28) divided by Equation (A29).

## Appendix D

Replace SIR with mass imbalance ratio (MIR) $a_{m}=1/a_{m}^{\prime }$ and the displacements of two coupled gyros are given as:

(A30) $$\omega_{1,2}^{2}=\frac{\left(1+a_{m}^{\prime }\right)\left(1+b\right)\pm \sqrt{{\left(1-a_{m}^{\prime }\right)}^{2}{\left(1+b\right)}^{2}+4a_{m}^{\prime }b^{2}}}{2m}k,\left(\omega_{1}<\omega_{2}\right),$$

(A31) $$v=\left[\begin{array}{c} v_{a1}+v_{F1}+v_{a2}+v_{F2}\\ I_{m1}v_{a1}+I_{m1}v_{F1}+I_{m2}v_{a2}+I_{m2}v_{F2} \end{array}\right]=\left[\begin{array}{c} v_{1}\\ v_{2} \end{array}\right],$$

(A32) $$I_{m1}=\frac{\left(1-a_{m}^{\prime }\right)\left(1+b\right)+\sqrt{{\left(1-a_{m}^{\prime }\right)}^{2}{\left(1+b\right)}^{2}+4a_{m}^{\prime }b^{2}}}{2b},$$

(A33) $$I_{m2}=\frac{\left(1-a_{m}^{\prime }\right)\left(1+b\right)-\sqrt{{\left(1-a_{m}^{\prime }\right)}^{2}{\left(1+b\right)}^{2}+4a_{m}^{\prime }b^{2}}}{2b},$$

(A34) $$v_{ai}=\frac{-1-I_{mi}a_{m}}{1+I_{mi}^{2}a_{m}}\frac{-a_{0}}{\omega_{i}^{2}\sqrt{{\left(1-\lambda_{ai}^{2}\right)}^{2}+{\left(2\xi_{i}\lambda_{ai}\right)}^{2}}}\sin\left(\omega_{v}t+\phi -\phi_{ai}\right)=\frac{-1-I_{mi}a_{m}}{1+I_{mi}^{2}a_{m}}v_{a0i},$$

(A35) $$v_{Fi}=\frac{1-I_{mi}}{1+I_{mi}^{2}a_{m}}\frac{F_{0}}{m\omega_{i}^{2}\sqrt{{\left(1-\lambda_{Fi}^{2}\right)}^{2}+{\left(2\xi_{i}\lambda_{Fi}\right)}^{2}}}\sin\left(\omega_{0}t-\phi_{Fi}\right)=\frac{1-I_{mi}}{1+I_{mi}^{2}a_{m}}v_{F0i},$$

(A36) $$v_{Fi}=\frac{1-I_{mi}a_{m}}{1+I_{mi}^{2}a_{m}}\frac{a_{co}}{\omega_{i}^{2}\sqrt{{\left(1-\lambda_{Fi}^{2}\right)}^{2}+{\left(2\xi_{i}\lambda_{Fi}\right)}^{2}}}\sin\left(\omega_{0}t-\phi_{Fi}\right)=\frac{1-I_{mi}a_{m}}{1+I_{mi}^{2}a_{m}}v_{F1i},$$

where $I_{m1}>0$ and $I_{m2}<0$ denote modal shape factors in the modal superposition method with mass imbalance whose absolute values are very near 1 and $I_{k1}I_{k2}=-a_{m}^{\prime }$, $\omega_{1}$ and $\omega_{2}$ are the resonant angular frequencies of the system, the frequency ratios $\lambda_{ai}=\frac{\omega_{v}}{\omega_{i}}$ and $\lambda_{Fi}=\frac{\omega_{0}}{\omega_{i}}$, the damping ratios $\xi_{i}≅\frac{(1+I_{mi}^{2})c}{2\left(1+a_{m}I_{mi}^{2}\right)m\omega_{i}}$, the phase angles $\phi_{ai}=\arctan\frac{2\xi_{i}\lambda_{ai}}{1-\lambda_{ai}^{2}},\phi_{Fi}=\arctan\frac{2\xi_{i}\lambda_{Fi}}{1-\lambda_{Fi}^{2}}$, i=1,2 . $v_{a0i}$ and $v_{F0i}$/$v_{F1i}$ are vibration and driving force/Coriolis-related displacement terms.

Next, the displacements difference is obtained (not the same for different internal forces):

driving forces:

(A37) $$\begin{array}{cc} v_{L}-v_{R}= & v_{e}+2v_{F02}=\frac{a_{m}I_{m1}^{2}+\left(1-a_{m}\right)I_{m1}-1}{1+a_{m}I_{m1}^{2}}v_{a01}+\frac{{\left(I_{m1}-1\right)}^{2}}{1+a_{m}I_{m1}^{2}}v_{F01}+\\ & \frac{a_{m}I_{m2}^{2}+\left(1-a_{m}\right)I_{m2}-1}{1+a_{m}I_{m2}^{2}}v_{a02}-\frac{\left(2a_{m}-1\right)I_{m2}^{2}+2I_{m2}+1}{1+a_{m}I_{m2}^{2}}v_{F02}+2v_{F02}. \end{array}$$

Similar to the stiffness imbalance when no vibration exists, the error displacements difference ratio is given by:

(A38) $$\rho_{m}≅\frac{{\left(I_{m2}-1\right)}^{2}}{2+2a_{m}I_{m2}^{2}}-1;$$

Coriolis forces:

(A39) $$\begin{array}{cc} v_{1}-v_{2}= & v_{e}+2v_{F02}=\frac{a_{m}I_{m1}^{2}+\left(1-a_{m}\right)I_{m1}-1}{1+a_{m}I_{m1}^{2}}v_{a01}+\frac{a_{m}I_{m1}^{2}-\left(1+a_{m}\right)I_{m1}+1}{1+a_{m}I_{m1}^{2}}v_{F01}\\ & +\frac{a_{m}I_{m2}^{2}+\left(1-a_{m}\right)I_{m2}-1}{1+a_{m}I_{m2}^{2}}v_{a02}-\frac{a_{m}I_{m2}^{2}+\left(1+a_{m}\right)I_{m2}+1}{1+a_{m}I_{m2}^{2}}v_{F02}+2v_{F02}. \end{array}$$

The error displacements difference ratio with no vibration is given by:

(A40) $$\rho_{m}≅-\frac{1}{2}-\frac{\left(1+a_{m}\right)I_{m2}}{2+2a_{m}I_{m2}^{2}}.$$

In the same way, when external vibration displacements are large enough compared to operating displacements, the error displacements difference $v_{e}$ in Equation (31) is approximately calculated by:

(A41) $$v_{e}≅\left(1+\frac{\left(1-a_{m}\right)I_{m1}-2}{{\left(1+\sqrt{a_{m}}I_{m2}\right)}^{2}-2\sqrt{a_{m}}I_{m2}}\right)v_{a02}≅\left(1-\frac{1-a_{m}}{2\sqrt{a_{m}}}+\frac{1}{\sqrt{a_{m}}I_{m2}}\right)v_{a02}.$$

With $\omega_{0}=\omega_{v}=\omega_{2}$ assumed:

(A42) $$v_{a02}=\frac{-a_{0}}{\omega_{2}^{2}2\xi_{2}}\cos\left(\omega_{2}t+\phi \right),$$

(A43) $$v_{F02}=\frac{-F_{d}}{2m\omega_{2}^{2}\xi_{2}}\cos\left(\omega_{2}t\right),v_{F12}=\frac{-a_{co}}{2\omega_{2}^{2}\xi_{2}}\cos\left(\omega_{2}t\right),$$

(A44) $$\begin{array}{c} v_{i}=2v_{F02}=\frac{-F_{d}}{m\omega_{2}^{2}\xi_{2}}\cos\left(\omega_{2}t\right),v_{i}=2v_{F12}=\frac{-a_{co}}{\omega_{2}^{2}\xi_{2}}\cos\left(\omega_{2}t\right), \end{array}$$

(A45) $$\begin{array}{c} v_{e}≅\left(1-\frac{1-a_{m}}{2\sqrt{a_{m}}}+\frac{1}{\sqrt{a_{m}}I_{m2}}\right)\frac{-a_{0}}{\omega_{2}^{2}2\xi_{2}}\cos\left(\omega_{2}t+\phi \right), \end{array}$$

where the frequency ratio $\lambda =\frac{\omega_{2}}{\omega_{1}}$, $\xi_{2}≅\frac{(1+I_{m2}^{2})c}{2\left(1+a_{m}I_{m2}^{2}\right)m\omega_{2}}$ is the damping ratio. Equation (18) is obtained by Equation (A45) divided by Equation (A44).

## Appendix E

The displacements of two uncoupled gyros with MIR $a_{m}$, $F_{L}=-F_{R}=F_{d}\sin\left(\omega_{0}t\right)$/$F_{L}=\frac{1}{a_{m}}F_{R}=ma_{co}\sin\left(\omega_{0}t\right)$ and $a_{v}=a_{0}\sin\left(\omega_{v}t+\phi \right)$ are given as (not the same for different internal forces):

(A46) $$\omega_{1}=\sqrt{\frac{k}{m}},\omega_{2}=\sqrt{\frac{k}{a_{m}m}};$$

driving forces:

(A47) $$v=\left[\begin{array}{c} \frac{-a_{0}\beta_{a1}}{\omega_{1}^{2}}\sin\left(\omega_{v}t+\phi -\phi_{a1}\right)+\frac{F_{0}\beta_{F1}}{m\omega_{1}^{2}}\sin\left(\omega_{0}t-\phi_{F1}\right)\\ a_{m}\frac{-a_{0}\beta_{a2}}{\omega_{1}^{2}}\sin\left(\omega_{v}t+\phi -\phi_{a2}\right)-\frac{F_{0}\beta_{F2}}{m\omega_{1}^{2}}\sin\left(\omega_{0}t-\phi_{F2}\right) \end{array}\right];$$

Coriolis forces

(A48) $$v=\left[\begin{array}{c} \frac{-a_{0}\beta_{a1}}{\omega_{1}^{2}}\sin\left(\omega_{v}t+\phi -\phi_{a1}\right)+\frac{a_{co}\beta_{F1}}{\omega_{1}^{2}}\sin\left(\omega_{0}t-\phi_{F1}\right)\\ a_{m}\frac{-a_{0}\beta_{a2}}{\omega_{1}^{2}}\sin\left(\omega_{v}t+\phi -\phi_{a2}\right)-a_{m}\frac{a_{co}\beta_{F2}}{\omega_{1}^{2}}\sin\left(\omega_{0}t-\phi_{F2}\right) \end{array}\right],$$

where $\beta_{ai}=\frac{1}{\sqrt{{\left(1-\lambda_{1i}^{2}\right)}^{2}+{\left(2\xi_{i}\lambda_{1i}\right)}^{2}}}$ and $\beta_{Fi}=\frac{1}{{\left(1-\lambda_{2i}^{2}\right)}^{2}+{\left(2\xi_{i}\lambda_{2i}\right)}^{2}}$ are the magnification factors of amplitude, $\omega_{1}$ and $\omega_{2}$ are the resonant angular frequencies of the system, the frequency ratios $\lambda_{ai}=\frac{\omega_{v}}{\omega_{i}}$ and $\lambda_{Fi}=\frac{\omega_{0}}{\omega_{i}}$, $\xi_{1}=\frac{c}{2m\omega_{1}}$ and $\xi_{2}=\frac{c}{2a_{m}m\omega_{2}}$ are the damping ratios, the phase angles $\phi_{ai}=\arctan\frac{2\xi_{i}\lambda_{ai}}{1-\lambda_{ai}^{2}}$, $\phi_{Fi}=\arctan\frac{2\xi_{i}\lambda_{Fi}}{1-\lambda_{Fi}^{2}}$, i=1,2.

If $\omega_{v}=\omega_{0}=\omega_{1}=\sqrt{a_{m}}\omega_{2}$, the displacements can be written as:

driving forces:
  (A49) $$v=\left[\begin{array}{c} \frac{a_{0}}{2\xi_{1}\omega_{1}^{2}}\cos\left(\omega_{1}t+\phi \right)-\frac{F_{0}}{2\xi_{1}m\omega_{1}^{2}}\cos\left(\omega_{1}t\right)\\ \frac{-a_{m}a_{0}}{J_{m}\omega_{1}^{2}}\sin\left(\omega_{1}t+\phi -\phi_{m}\right)-\frac{F_{0}}{mJ_{m}\omega_{1}^{2}}\sin\left(\omega_{1}t-\phi_{m}\right) \end{array}\right]=\left[\begin{array}{c} v_{L}\\ v_{R} \end{array}\right];$$
Coriolis forces:
  (A50) $$v=\left[\begin{array}{c} \frac{a_{0}}{2\xi_{1}\omega_{1}^{2}}\cos\left(\omega_{1}t+\phi \right)-\frac{a_{c0}}{2\xi_{1}m\omega_{1}^{2}}\cos\left(\omega_{1}t\right)\\ \frac{-a_{m}a_{0}}{J_{m}\omega_{1}^{2}}\sin\left(\omega_{1}t+\phi -\phi_{m}\right)-\frac{a_{m}a_{co}}{mJ_{m}\omega_{1}^{2}}\sin\left(\omega_{1}t-\phi_{m}\right) \end{array}\right]=\left[\begin{array}{c} v_{L}\\ v_{R} \end{array}\right],$$
  (A51) $$J_{m}=\frac{1}{\xi_{1}}\sqrt{{\left(1-\frac{\omega_{1}^{2}}{\omega_{2}^{2}}\right)}^{2}+{\left(2\xi_{2}\frac{\omega_{1}}{\omega_{2}}\right)}^{2}}=\sqrt{{\left(a_{m}-1\right)}^{2}/\xi_{1}^{2}+4}.$$

The displacements difference can be written as:

driving forces:
  (A52) $$v_{L}-v_{R}≅\left(\frac{1}{2\xi_{1}}-\frac{a_{m}}{J_{m}\xi_{1}}\sin\phi_{m}\right)\frac{a_{0}}{\omega_{1}^{2}}\cos\left(\omega_{1}t\right)-\left(\frac{1}{2\xi_{1}}+\frac{1}{J_{m}\xi_{1}}\sin\phi_{m}\right)\frac{F_{0}}{m\omega_{1}^{2}}\cos\left(\omega_{1}t\right);$$
Coriolis forces
  (A53) $$v_{L}-v_{R}≅\left(\frac{1}{2\xi_{1}}-\frac{a_{m}}{J_{m}\xi_{1}}\sin\phi_{m}\right)\frac{a_{0}}{\omega_{1}^{2}}\cos\left(\omega_{1}t\right)-\left(\frac{1}{2\xi_{1}}+\frac{a_{m}}{J_{m}\xi_{1}}\sin\phi_{m}\right)\frac{a_{co}}{\omega_{1}^{2}}\cos\left(\omega_{1}t\right),$$
  where the phase angle $\phi_{m}=\arctan\frac{2\xi_{1}\sqrt{a_{m}}}{1-a_{m}},\xi_{1}=\frac{c}{2m\omega_{1}}$ is the damping ratio. Ideal displacements difference $v_{i}=\frac{-F_{d}}{m\omega_{2}^{2}\xi_{2}}\cos\left(\omega_{2}t\right)$/ $v_{i}=\frac{-a_{co}}{\omega_{2}^{2}\xi_{2}}\cos\left(\omega_{2}t\right)$ and error displacements difference ratio is given by $\left(v_{L}-v_{R}-v_{i}\right)/v_{i}$. Therefore, Equations (23) and (24) are achieved.

## Appendix F

The dynamics of coupled gyros with damping imbalance are derived. For two DOFs system with coupled gyros with damping imbalance ratio (DIR) $a_{c}$, Equation (3) can be transferred to Laplace form:

(A54) $$\left[\begin{array}{cc} ms^{2}+cs+\left(1+b\right)k & -bk\\ -bk & ms^{2}+a_{c}cs+\left(1+b\right)k \end{array}\right]\left[\begin{array}{c} V_{L}\left(s\right)\\ V_{R}\left(s\right) \end{array}\right]=\left[\begin{array}{c} A(s)+F(s)\\ A(s)-F(s) \end{array}\right].$$

The solution through Cramer’s Rule is given by:

(A55) $$\begin{array}{c} \left[\begin{array}{c} V_{L}\left(s\right)\\ V_{R}\left(s\right) \end{array}\right]=\left[\begin{array}{c} \frac{ms^{2}+a_{c}cs+\left(1+2b\right)k}{D(s)}A\left(s\right)+\frac{ms^{2}+a_{c}cs+k}{D(s)}F\left(s\right)\\ \frac{ms^{2}+cs+\left(1+2b\right)k}{D(s)}A\left(s\right)-\frac{ms^{2}+cs+k}{D(s)}F\left(s\right) \end{array}\right], \end{array}$$

(A56) $$\begin{array}{c} V_{L}\left(s\right)-V_{R}\left(s\right)=\frac{(a_{c}-1)cs}{D(s)}\left(A\left(s\right)-F\left(s\right)\right)+\frac{ms^{2}+2cs+k}{D(s)}F\left(s\right)=E\left(s\right)+V_{i}\left(s\right). \end{array}$$

Here, s=jω, $A\left(s\right),F\left(s\right),V_{L}\left(s\right)$, and $V_{R}\left(s\right)$ are Laplace transforms of $a,F_{L},v_{L}$, and $v_{R}$. $D\left(s\right)=m^{2}s^{4}+\left(1+a_{c}\right)cs^{3}+\left(2\left(b+1\right)mk+a_{c}c^{2}\right)s^{2}+\left(1+a_{c}\right)\left(1+b\right)ks+\left(1+2b\right)k^{2},V_{i}$ is the ideal TFG’s displacements difference. $(a_{c}-1)\ll 2$ is easy to find. Hence, the error outputs are very small compared with other situations.

## Appendix G

For an uncoupled two-gyro system with $a_{c}$, the displacements of two gyros:

(A57) $$v=\left[\begin{array}{c} \frac{-a_{0}\beta_{a1}}{\omega^{2}}\sin\left(\omega_{v}t+\phi -\phi_{a1}\right)+\frac{F_{0}\beta_{F1}}{m\omega^{2}}\sin\left(\omega_{0}t-\phi_{F1}\right)\\ \frac{-a_{0}\beta_{a2}}{\omega^{2}}\sin\left(\omega_{v}t+\phi -\phi_{a1}\right)-\frac{F_{0}\beta_{F2}}{m\omega^{2}}\sin\left(\omega_{0}t-\phi_{F2}\right) \end{array}\right]=\left[\begin{array}{c} v_{L}\\ v_{R} \end{array}\right],$$

where $\beta_{ai}=\frac{1}{\sqrt{{\left(1-\lambda_{ai}^{2}\right)}^{2}+{\left(2\xi_{i}\lambda_{ai}\right)}^{2}}}$ and $\beta_{Fi}=\frac{1}{\sqrt{{\left(1-\lambda_{Fi}^{2}\right)}^{2}+{\left(2\xi_{i}\lambda_{Fi}\right)}^{2}}}$ denote the magnification factors of amplitude, $\phi_{ai}=\arctan\frac{2\xi_{i}\lambda_{ai}}{1-\lambda_{ai}^{2}}$ and $\phi_{Fi}=\arctan\frac{2\xi_{i}\lambda_{Fi}}{1-\lambda_{Fi}^{2}}$ are the phase angles, the damping ratios $\xi_{1}=\frac{c}{2m\omega }$ and $\xi_{2}=\frac{a_{c}c}{2m\omega }$, i=1,2, and the resonant angular frequency $\omega =\sqrt{\frac{k}{m}}$.

If $\omega_{v}=\omega_{0}=\omega$, the displacements difference and error/ideal displacements difference can be expressed as:

(A58) $$v_{L}-v_{R}=\left(1-\frac{1}{a_{c}}\right)\frac{a_{0}}{2\xi_{1}\omega^{2}}\cos\left(\omega t+\phi \right)-\left(1+\frac{1}{a_{c}}\right)\frac{F_{0}}{2m\xi_{1}\omega^{2}}\cos\left(\omega t\right),$$

(A59) $$v_{e}=\left(1-\frac{1}{a_{c}}\right)\frac{a_{0}}{2\xi_{1}\omega^{2}}\cos\left(\omega t+\phi \right)+\left(\frac{1}{2}-\frac{1}{2a_{c}}\right)\frac{-F_{0}}{m\xi_{1}\omega^{2}}\cos\left(\omega t\right),v_{i}=\frac{-F_{0}}{m\xi_{1}\omega^{2}}\cos\left(\omega t\right).$$

From Equations (A59) and (25) is obtained.
